# Supplementary material for: PfCERLI1 is a conserved rhoptry associated protein essential for Plasmodium falciparum merozoite invasion of erythrocytes
Source: Nat Commun. 2020 Mar 16;11:1411. doi: 10.1038/s41467-020-15127-w (PMC7075938; doi:10.1038/s41467-020-15127-w)
Supplement: Supplementary file 3 — Reporting Summary [file 41467_2020_15127_MOESM3_ESM.pdf]

nature research

Corresponding author(s): Danny Wilson

Last updated by author(s): Jan 29, 2020

Reporting Summary

Nature Research wishes to improve the reproducibility of the work that we publish. This form provides structure for consistency and transparency in reporting. For further information on Nature Research policies, seeAuthors & Referees and theEditorial Policy Checklist .

Statistics

For all statistical analyses, confirm that the following items are present in the figure legend, table legend, main text, or Methods section.

n/a	Confirmed	
☐	☒	The exact sample size (*n*) for each experimental group/condition, given as a discrete number and unit of measurement
☐	☒	A statement on whether measurements were taken from distinct samples or whether the same sample was measured repeatedly
☐	☒	The statistical test(s) used AND whether they are one- or two-sided<br>*Only common tests should be described solely by name; describe more complex techniques in the Methods section.*
☒	☐	A description of all covariates tested
☒	☐	A description of any assumptions or corrections, such as tests of normality and adjustment for multiple comparisons
☐	☒	A full description of the statistical parameters including central tendency (e.g. means) or other basic estimates (e.g. regression coefficient) AND variation (e.g. standard deviation) or associated estimates of uncertainty (e.g. confidence intervals)
☒	☐	For null hypothesis testing, the test statistic (e.g. *F*, *t*, *r*) with confidence intervals, effect sizes, degrees of freedom and *P* value noted<br>*Give P values as exact values whenever suitable.*
☒	☐	For Bayesian analysis, information on the choice of priors and Markov chain Monte Carlo settings
☒	☐	For hierarchical and complex designs, identification of the appropriate level for tests and full reporting of outcomes
☐	☒	Estimates of effect sizes (e.g. Cohen's *d*, Pearson's *r*), indicating how they were calculated

Our web collection on statistics for biologists contains articles on many of the points above.

Software and code

Policy information about availability of computer code

Data collection	Super-resolution microscopy: Zeiss LSM800 with Airyscan<br>Confocal microscopy: Olympus FV3000<br>Western blot imaging: Li-COR Odyssey<br>Flow cytometer: BD LSR II<br>Signal peptide prediction: SignalP-3.0 & SignalP-5.0<br>Structure prediction: Phyre2 & I-TASSER.
Data analysis	Microscopy image processing and analysis: Imaris version 9<br>Statistical analysis and graph generation: PRISM version 8<br>Measuring of rhoptry bulb diameter: ZEN Blue(VERSION?)<br>Pairwise distance calculation: Geneious version 9.1.3<br>Analysis of flow cytometry data: FlowJo Version 10<br>Quantification of Western blots: ImageStudioLite Version 5.2.5

For manuscripts utilizing custom algorithms or software that are central to the research but not yet described in published literature, software must be made available to editors/reviewers. We strongly encourage code deposition in a community repository (e.g. GitHub). See the Nature Research guidelines for submitting code & software for further information.

Data

Policy information about availability of data

All manuscripts must include a data availability statement. This statement should provide the following information, where applicable:

- Accession codes, unique identifiers, or web links for publicly available datasets
- A list of figures that have associated raw data
- A description of any restrictions on data availability

All data available upon request.

Field-specific reporting

Please select the one below that is the best fit for your research. If you are not sure, read the appropriate sections before making your selection.

[x] Life sciences   [ ] Behavioural & social sciences   [ ] Ecological, evolutionary & environmental sciences

For a reference copy of the document with all sections, see nature.com/documents/nr-reporting-summary-flat.pdf

Life sciences study design

All studies must disclose on these points even when the disclosure is negative.

Sample size

Figure 1d: Representative image shown
Figure 1e: Representative image shown
Figure 1f: Representative image shown
Figure 1g: >50,000 cells counted for each sample.
Figure 2a: >50,000 cells counted for each sample.
Figure 2b: 20 schizonts counted for each biological replicate.
Figure 2c: >500 late stages counted for each replicate
Figure 2d: >1900 mean number of free merozoites counted per replicate
Figure 2e: Representative image shown
Figure 3a: Representative images shown
Figure 3b: Sample size per marker = 18
Figure 3c: Representative image shown
Figure 4a-d: Representative images shown
Figure 4e: >1000 rhoptries measured for each marker
Figure 4f: Representative image shown
Figure 4g: Representative image shown
Figure 5a: Representative images shown
Figure 5b-d: Total sample size = 38 for untreated, 35 for + GLCN
Figure 5e: >1200 rhoptries measured for each treatment
Figure 6a: Representative image shown
Supplementary Figure 1b: Representative images shown
Supplementary Figure 1c: Sample size = 3 for each marker
Supplementary Figure 2a: >50,000 events counted for each sample
Supplementary Figure 2b: >50,000 events counted for each sample
Supplementary Figure 2c: >1900 mean number of free merozoites counter per replicate
Supplementary Figure 4c: Representative images shown
Supplementary Figure 5: Representative images shown
Supplementary Figure 6: >1000 signals quantified per marker, per treatment
Supplementary Figure 7: Total sample size = 19 untreated, 21 for +GLCN
Supplementary Figure 8: >1000 signals quantified per treatment

Data exclusions

No data were excluded

Replication

Figure 1 e,f: Blots performed in biological triplicate
Figure 1g: Performed in biological triplicate
Figure 2a&d: Performed in biological quadruplicate
Figure 2b: Performed in biological triplicate
Figure 2c: Performed in biological triplicate
Figure 3b: Performed in biological triplicate, 6 images per replicate
Figure 4 a,b: Blots performed in biological triplicate
Figure 4e: Data collected from 5 biological replicates
Figure 5: Data from all experiments collected from 5 biological replicates
Figure 6: Data collected and quantified from 5 biological replicates

	Supplementary Figure 1: Blots performed in biological triplicate<br>Supplementary Figure 2a: Performed in biological triplicate<br>Supplementary Figure 2b: Performed in biological duplicate<br>Supplementary Figure 2c: Data interpreted from Figure 2d, which was performed in biological quadruplicate<br>Supplementary Figure 6,7 & 8: Data collected from 5 biological replicates<br>Supplementary Figure 9a: Data collected and quantified from 5 biological replicates<br>Supplementary Figure 9b&c: Data collected and quantified from 4 biological replicates
Randomization	N/A
Blinding	Figure 2b: Giemsa stained smears in biological triplicate were blinded. Merozoites per schizont was counted by microscopy while blinded.

Reporting for specific materials, systems and methods

We require information from authors about some types of materials, experimental systems and methods used in many studies. Here, indicate whether each material, system or method listed is relevant to your study. If you are not sure if a list item applies to your research, read the appropriate section before selecting a response.

Materials & experimental systems

n/a	Involved in the study
☐	☒ Antibodies
☒	☐ Eukaryotic cell lines
☒	☐ Palaeontology
☒	☐ Animals and other organisms
☒	☐ Human research participants
☒	☐ Clinical data

Methods

n/a	Involved in the study
☒	☐ ChIP-seq
☐	☒ Flow cytometry
☒	☐ MRI-based neuroimaging

Antibodies

Antibodies used	Commercial antibodies:<br>Mouse anti-HA (12CA5), Roche, 11583816001<br>Chicken anti-HA, Abcam, ab9111<br>Goat anti-Mouse IgG IRDye 800CW, Li-COR Biosciences, 926-32210<br>Goat anti-Rabbit IgG IRDye 680RD, Li-COR Biosciences, 926-68071<br>Alexa Fluor 488 Goat anti-Chicken IgY, Life Technologies, A-11039<br>Alexa Fluor 594 Goat anti-Mouse IgG H+L, Life Technologies, A-11005<br>Alexa Fluor 647 Goat anti-Rabbit IgG H+L, Life Technologies, A-21245<br><br>Non-commercial antibodies:<br>Primary antibodies mouse anti-CyRPA (8A7), mouse anti-RH5, mouse anti-RAP1 rabbit anti-RON4, rabbit anti-EBA175 and rabbit anti-GAP45(R728K) were provided by Prof. Alan Cowman at the Walter and Eliza Hall Institute of Medical Research. Rabbit anti-EXP2 was provided by Dr. Paul Gilson at the Burnet Institute. Rabbit anti-ERC and rabbit anti-GAPDH were provided by Prof. Leann Tilley at Bio21 Institute.
Validation	Antibodies previously validated commercially or by the laboratories listed in peer reviewed publications (available in manuscript).

Flow Cytometry

Plots

Confirm that:

- ☒ The axis labels state the marker and fluorochrome used (e.g. CD4-FITC).
- ☒ The axis scales are clearly visible. Include numbers along axes only for bottom left plot of group (a 'group' is an analysis of identical markers).
- ☒ All plots are contour plots with outliers or pseudocolor plots.
- ☒ A numerical value for number of cells or percentage (with statistics) is provided.

Methodology

Sample preparation	For growth assays: Cells were washed once in PBS, before staining in PBS + Ethidium bromide for 30 minutes. Cells were then washed twice with PBS before flow cytometry analysis.<br>For invasion assays: To 50 microlitres of cells, 170 microlitres of PBS + Ethidium bromide was added. Cells were stained for 30 minutes in the dark before flow cytometry analysis.

Instrument	Becton Dickinson LSR II
Software	FlowJo version 10
Cell population abundance	For all flow cytometry experiments, between approximately 50,000 and 80,000 events were captured. Of these, typically >90% fell inside the red blood cell FSC/SSC gate. For growth and invasion assays, trophozoite and ring stage populations typically numbered between 1000 and 10000 events, depending on the parasiteaemia of that treatment. For counting free merozoites by flow cytometry, typically between 2000 and 10000 events were captured, again depending on the parasitaemia of that treatment.
Gating strategy	Red blood cell populations were gated by FSC and SSC. Within this RBC population, newly invaded ring-stage parasites were gated on intermediate FITC (GFP) fluorescence and low PE (Ethidium bromide) fluorescence, late-stage parasites were gated as PE (Ethidium bromide) high events. Free merozoites were gated as FITC (GFP) high and PE (ethidium bromide) high events that occurred outside (lower FSC/SSC) of the red blood cell gate.

☒ Tick this box to confirm that a figure exemplifying the gating strategy is provided in the Supplementary Information.
